# Virtual Reality experiments in the field

**DOI:** 10.1371/journal.pone.0318688

**Published:** 2025-04-08

**Authors:** Maria Alejandra Quirós-Ramírez, Anna Feineisen, Stephan Streuber, Ulf-Dietrich Reips

**Affiliations:** 1 Department of Psychology, University of Konstanz, Konstanz, Baden-Württemberg, Germany; 2 Department of Electrical Engineering and Computer Science, Coburg University of Applied Sciences, Coburg, Bavaria, Germany; University of Lahore - Raiwind Road Campus: The University of Lahore, PAKISTAN

## Abstract

Virtual Reality (VR) has paved its way into experimental psychology due to its capacity to realistically simulate real-world experiences in a controlled way. Theoretically, this technology opens the possibility to conduct experiments anywhere in the world using consumer hardware (e.g. mobile-VR). This would allow researchers to access large scale, heterogeneous samples and to conduct experiments in the field in cases where social distancing is required – e.g. during the COVID-19 pandemic. Here, we investigate the feasibility of carrying VR experiments in the field using mobile-VR through a stress inductive (public speaking task) and a relaxation (nature) task and contrast them with results in the laboratory (HTC Vive and mobile-VR). The first experiment employed a 2 (device: HTC Vive Pro (HMD) versus Wearality Sky VR smartphone adapter) x 3 (audience: ‘none’, ‘attentive’, ‘inattentive’) between-subjects design. Thirty-four participants took part in the experiment and completed a public speaking task. No significant difference was detected in participants’ sense of presence, cybersickness, or stress levels. In the second experiment, using an inexpensive Google Cardboard smartphone adapter a 3 (between: device setting) x 2 (within: task) mixed-design was employed. Sixty participants joined the experiment, and completed a public speaking and a nature observation task. No significant difference in participants’ sense of presence, cybersickness, perceived stress and relaxation were detected. Taken together, our results provide initial evidence supporting the feasibility and validity of using mobile VR in specific psychological field experiments, such as stress induction and relaxation tasks, conducted in the field. We discuss challenges and concrete recommendations for using VR in field experiments. Future research is needed to evaluate its applicability across a broader range of experimental paradigms.

## Introduction

Behavioral research using Virtual Reality (VR) has expanded exponentially in the last decades [1–3]. VR provides multiple benefits to experimental research, including an increased experimental control and ecological validity [4,5], as well as decreasing experimental costs, increasing portability, and easing the exchange of experimental set-ups between researchers. Research in VR spans a wide variety of psychological fields and topics, from eating disorders [14,15], social anxiety and stress [16], addictions [17], racism [18], all the way to marketing [19]. VR has been widely applied in clinical environments, such as in VR exposure therapy (VRET) for treating fears of heights, flying [20] and social phobias [21]. It has also been utilized for psychological assessment, including body image [22], psychosis [23], and emotions [24], and is considered pivotal for advancing the psychotherapy of eating and weight disorders [25].

There are, however, situations in which it is not possible to access participants for experiments in the laboratory, for example the Covid-19 pandemic and its restrictions. Bringing VR technologies outside of the laboratory would enable scientists to (a) access to populations that would not be accessible due to physical and geographical constraints, (b) generally bring VR experiences to ecologically valid settings, i.e. people’s homes, (c) enable access to a wide and diverse participant pool, and (d) keep conducting studies in circumstances where face-to-face activities are not possible to carry out normally – like the Covid-19 crisis. Even though, there are different resources for running experiments online [6–13], these web options were not made for VR research, thus, they are rarely suitable for carrying out VR experiments.

Is it possible to reliably conduct VR experiments in the field? Here, we investigate whether mobile-VR is a reliable alternative to conducting VR experiments outside of the laboratory. For this purpose we carried out two experiments. Experiment 1 investigates the effect of VR equipment (high-end VR vs. mobile VR) on behavioral and physiological outcomes of a psychological experiment. Experiment 2 investigates the effect of the experimental environment (laboratory vs. field) and device (high-end VR vs. mobile VR) on the behavioral outcomes. The results of these experiments will provide insights into the effectiveness and feasibility of using mobile VR equipment for specific types of psychological experiments conducted in field settings.

Conducting VR experiments outside of the laboratory poses multiple challenges with regards to equipment management and experimental control. The laboratory provides a controlled and safe environment void of possible distractors, such as noises or people entering the room, and typically counts with the physical presence of an experimenter, who monitors the experiment, provides a safety, and serves as a contact point for questions. Another challenge is the availability of VR equipment outside the laboratory. Given the dimensions and costs of the high-end VR equipment typically used in the laboratory, it would be difficult, and perhaps not recommended in most cases, to deliver it to participants outside of the laboratory. To curb these shortcomings, the following experimental decisions were made: (1) an experimenter would be virtually available through a videoconferencing platform to enhance experimental control and (2) using mobile VR with the participants’ own smartphones instead of the laboratory’s high-end VR equipment.

We expect to see similar results in the measures of performance and subjective experience of the participants in the tasks in the laboratory and in the field. However, we expect the laboratory condition with the high-end VR headset to score better in terms of *immersion* and *presence* and cause less *cybersickness* in participants (see the Virtual Reality section for details). If we can determine that (a) virtual environments elicit similar responses in high-end commercial VR equipment and low-cost mobile VR equipment, and that (b) these responses are similar if the experiment is conducted in the laboratory as well as in the field, this would provide promising evidence for the feasibility of using mobile VR in field experiments. Importantly, if none of our results speak against using VR in the field, the combination of low-cost devices and remote support via video calls could expand opportunities for studying behavior in situ under varied conditions.

## Virtual reality

Virtual Reality refers to a three-dimensional (3D) computer simulation of an artificial world, also known as *virtual environment* (VE). This simulation provides a multisensory experience for the user, based on action-perception loops achieved through tracking and display hardware. Based on information obtained from tracking devices placed on the user, such as head position, the computer simulation is updated to provide specific sensorial feedback for the user through different types of displays, such as visual or olfactory displays, giving the user the illusion of *being* inside this artificial 3D world [26]. Currently, the most widespread VR devices are called *head mounted displays* (HMD). These can track, at least, the user’s head rotation and can include extra trackers to be placed in different parts of the body, as well as hand controllers for interactivity, while providing visual and auditory feedback to the user.

There is a particular feature that characterizes VR: *presence*. Presence is a psychological construct that refers to the subjective experience or illusion of ‘being there’ in the virtual environment [26]. A high level of presence leads to a high level of behavioural response realism—users respond similarly in virtual and real environments [27]. Presence can be measured through questionnaires [26]. It is usually utilized as a measure of success and quality of a virtual environment [28].

One downside of VR is the proneness of some users to suffer sickness symptoms similar to those of motion sickness, for example headaches or queasiness. Cybersickness in VR can be caused by multiple reasons, such as a discrepancy or temporal delay between visual and vestibular information [29].

VR research carried out before the 2010s relied exclusively on specialized, very expensive, and bulky technology. The boom of consumer VR around the year 2014 [26] significantly improved accessibility to the technology, enabling a larger number of research laboratories to adopt it. However, there are still multiple drawbacks and constraints preventing the use of VR in a similar manner as psychologists use computers nowadays. Namely, (a) specialized programming knowledge is required to create the VR environments, (b) even though the technology has become less expensive in comparison to its previous versions, a robust VR set-up can be outside of the budget of the average laboratory, (c) a moderate degree of expertise is still necessary to set-up and manage the hardware, ensure user experience, and provide proper guidance.

**Fig 1:**
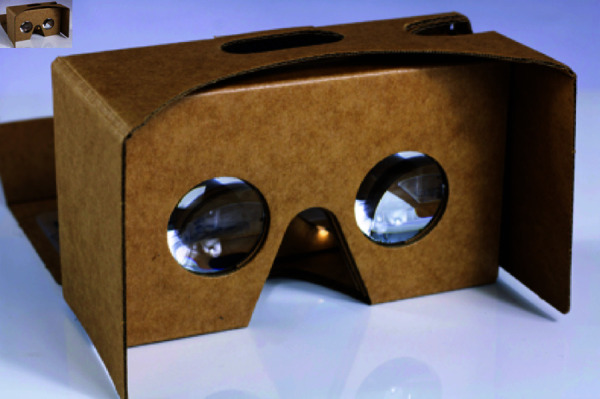
Google cardboard. Low-cost Virtual Reality smartphone adapter made out of cardboard, which can be purchased as a pre-manufactured viewer or built by users following specifications published by Google.

A plausible solution to some of these constraints may be the use of *mobile VR*. Mobile VR refers to the use of a consumer-grade smartphone to run VR environments and tasks, together with a special adapter to convert the smartphone into a head mounted display (HMD). There are multiple adapter options, ranging from very sophisticated hardware, like Samsung Gear VR, which visually resembles the high-end HMDs, to the lowest-cost available one: Google Cardboard (Fig 1). Unlike Samsung Gear VR – which is only compatible with a subset of Samsung smartphones – Google Cardboard supports a large variety of smartphones, including the iPhone. It is also possible to build a Cardboard following Google’s specifications with home-owned materials, or to purchase pre-manufactured ones (with bulk prices as low as $0,05 per unit, as of 2024).

Up to date, a handful of studies report using VR outside of the laboratory. Steed et al. [30] conducted a Human-Computer Interaction (HCI) experiment aimed at developing design guidelines for future consumer VR applications. The study probes the VR features of *presence* and *embodiment* through a simple observation task with avatars. Participants could join if they owned a Google Cardboard or Samsung Gear VR. As the authors mention, their study is uncontrolled, and there is no comparison with laboratory results. Then, Mottelson and Hornæk [31] developed a different Human Computer Interaction (HCI) study to investigate pointing, 3D tracing, and body-illusions in the laboratory (HTC Vive) and in the field (Google Cardboard), with a follow-up task in the laboratory using Google Cardboard. Their results show the potential of empirical HCI studies using VR outside of the laboratory. Finally, Mottelson et al. [32], Radiah et al. [35], Huber and Gajos [33], Saffo et al. [34], and Steed et al. [30] tested different tasks with consumer-owned devices, showcasing the possibility of recruiting VR device owners and replicating previous results from the laboratory. In a recent survey, Ratcliffe et al. [36] outlined the series of possibilities and challenges potentially presented by the use of VR in experiments outside of the lab. However, whether mobile VR is feasible for field experiments in Psychology remains an open question.

## Experiment 1

We first investigated the effect of using a low-end mobile VR for experiments as an alternative to the usual high-end VR. For this purpose, we designed and developed a stress inductive, public speaking VE where participants were asked to interact with a virtual crowd by giving an impromptu speech, inspired by existing stress induction paradigms in VR (e.g. the VR Trier Social Stress Test by Zimmer et al. [37]).

### Method

#### Materials.

**Fig 2:**
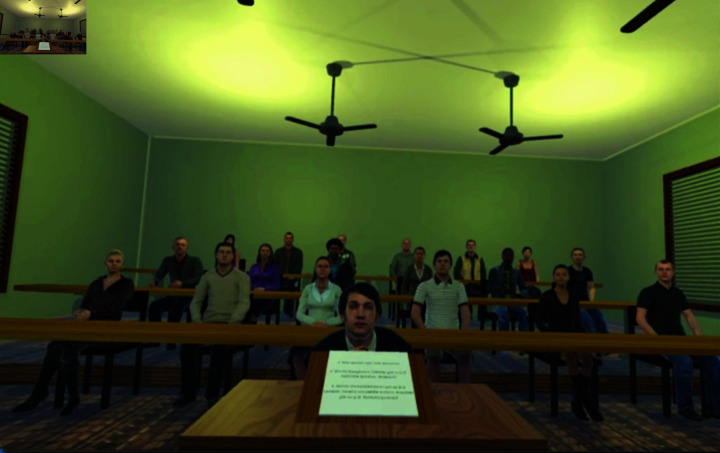
Public speaking VR experience. The participant stands in front of the lectern, which has the talking points for the impromptu speech.

*Virtual Environment*. The VE for Experiment 1 is a computer-generated classroom, where the participant acts as a speaker, standing behind a lectern (see Fig 2). Participants do not see their bodies in the VE. The speech topic is “Evolution”. The virtual classroom contains, on top of the lectern, a note with three written key points for the speech that reads: *What is evolution?*, *Which kinds of biological factors exist (e.g., natural selection, mutation)?*, and *What theories of evolution (e.g., Lamarck, Darwin) and which other perspectives (like creationism) are known?*

The room has moving ceiling ventilators, which make some noise, an emergency exit behind the speaker, and three rows of seats in front of the speaker. Additionally, there are three single seats placed very close to the lectern.

There is a virtual audience seated in front of the participants that can be either ‘attentive’ (follows the participant’s head with their gaze) or ‘inattentive’ (purposely looks away from the participant’s head). We included a control condition, ‘none’, where the classroom is empty.

**Fig 3:**
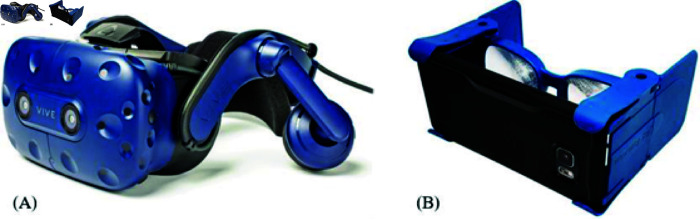
HTC Vive Pro headset and wearality sky. (A) HTC Vive Pro headset, (B) Wearality Sky, smartphone VR adapter.

*Apparatus*. As a high-end commercial VR device, we utilized the HTC Vive Pro HMD (Fig 3A) powered by a VR ready PC (VR One 6RE, msi, Taipei Taiwan; CPU: Intel® Core™ i7 processor, 6th generation; Graphics card: GeForce® GTX 1070 with 8 GB GDDR5). The Vive Pro has a resolution of 1440 x 1600 pixels per eye, a refresh rate of 90 Hz, and a 110° FOV. The sound was provided by Hi-Res Certified™ on-ear headphones, which are part of the HMD. Two base stations were placed in the lab to provide a tracking area of approximately 3 x 2 m for the participants.

As a low-end mobile VR, we utilized the Wearability Sky, a VR smartphone adapter with double Fresnel lenses and 150° FOV (Fig 3B). The VE was presented on a smartphone (ONEPLUS 3, OnePlus, Shenzhen People’s Republic of China) with 6 GB RAM, a resolution of around 540 x 960 pixels per eye, 60 Hz refresh rate and a display size of 5.5’’. Sound was provided with on-ear headphones (E45BT, JBL, Los Angeles United States).

#### Design.

The experiment was conducted in a 2 (device) x 3 (audience) between-subjects design. The device levels were HTC Vive Pro (HMD) and Wearality Sky (VR smartphone adapter), and the audience levels were ‘none’, ‘attentive’, ‘inattentive’. Participants were randomly assigned to one of the different experimental conditions. Questionnaires were provided in German using paper-pencil questionnaires.

**Table 1:** HR intervals and corresponding events.

HR Interval	Time (min)	Event
*t*_0_	0-5	Baseline stress rating
*t*_1_	5-7	Instructions
*t*_2_	7-10	Pre-task stress rating
*t*_3_	10-11.5	Prepare for VR
*t*_4_	11.5-16.5	VR task
*t*_5_	16.5-18.5	Post-task stress rating
*t*_6_	18.5-30	Recovery
*t*_7_	30-31	Final stress rating

#### Measures.

*Visual Analogue Scale (VAS)*. Self-reported stress was measured by using the Visual Analogue Scale [6] in a pre-task (from here on: VAS-A) and a post-task (from here on: VAS-B) version. In the VAS-A, participants had to rate two statements: “I feel stressed." and “I feel strained." on a line between “do not agree” and “agree” (0-100, no numbers visible to participants). In the VAS-B, three items were included to evaluate the source of stress, in addition to the VAS-A statements: “Talking was particularly stressful.", “The (in-) attentiveness of the audience was particularly stressful.” and “To be immersed in a virtual environment was particularly stressful.” Ratings were collected at four time points: at the beginning ($t_{0-VAS}$, baseline; VAS-A), after the task instructions ($t_{1-VAS}$, instructions; VAS-A), immediately after the task ($t_{2-VAS}$, post-task; VAS-B), and at the end ($t_{3-VAS}$, recovery; VAS-B).

**Fig 4:**
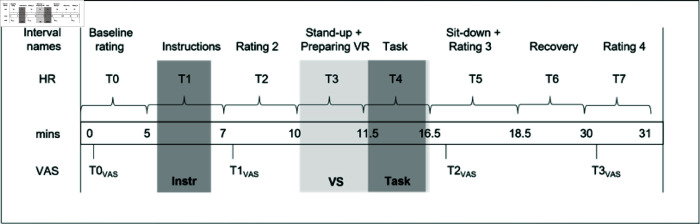
Procedure Overview for Experiment 1 (Chronological Order). HR: heart rate, VAS: Visual Analogue Scale.

*Heart Rate (HR)*. Heart rate (HR), a physiological stress marker, was continuously measured throughout the experiment using a strap-based device (Polar OH1, Polar Electro, Büttelborn, Germany) worn on the left forearm. For the evaluation and comparison, the HR measurement was subdivided into eight distinct intervals for evaluation and comparison, with the arithmetic mean, minimum, and maximum HR calculated for each interval. The HR intervals are shown in Table 1 and in Fig 4.

*iGroup Presence Questionnaire (IPQ)*. The sense of presence was measured using the German version of the iGroup Presence Questionnaire (IPQ) [38]. It is comprised of 13 items assessing three subscales, (a) *Spatial Presence*, the sense of being physically present in the VE, (b) *Involvement*, the dedicated attention to the VE as well as the experienced involvement, and (c) *Experienced Realism*, the subjective experience of realism in the VE. Additionally, IPQ includes one item to assess the overall *Sense of Being There* in the VE. Each item is rated on a 7-point Likert scale with varying anchors, where higher values indicate a greater sense of presence. The IPQ has demonstrated good internal consistencies, with Cronbach’s *α* ranging from .85 to 0.87 [38].

*Cybersickness*. Cybersickness was evaluated using a simplified symptom checklist adapted from the Simulator Sickness Questionnaire (SSQ) by Lane and Kennedy [39]. The checklist included five dichotomous (“yes”/“no”) items for symptoms commonly associated with VR: vertigo, nausea, headache, general discomfort, and fatigue. These symptoms were drawn from the three dimensions of the SSQ (Nausea, Oculomotor, and Disorientation). Participants could also report additional symptoms if experienced. If one or more symptoms were reported, participants answered two follow-up questions to attribute the symptoms to either performing the task or being immersed in VR. Cybersickness was considered present if symptoms were attributed to VR immersion. This simplified assessment was chosen to reduce participant burden and was deemed sufficient given the limited movement and interaction in the VEs, which were designed to minimize temporal delay. As a result, the overall risk of severe cybersickness was expected to be low.

*Previous experience with VR*. Participants’ previous experience with VR was recorded by asking participants to rate how often they have used VR in the past, selecting between the five possible options: “never", “1 to 5 times", “6 to 10 times", “11 to 15 times", “more than 15 times."

#### Procedure.

The experiment was conducted in accordance with the ethical standards of the University of Konstanz and with the Helsinki Declaration. The Ethics Committee (institutional review board, IRB) of the University of Konstanz issued a waiver for our experiments (RefNo: IRB24KN05-008/w). All participants gave written informed consent before taking part in the study. Participants were rewarded for their participation and they were explicitly made aware that they were allowed to withdraw from the study at any point in time, without losing their reward nor affecting the study.

Participants arrived at the laboratory and were given information about the experiment (risks and benefits, data acquisition, etc.). They were requested to give written informed consent in order to take part in the experiment. For HR measurement, the strap was placed on the participant’s left arm and instructions on how to complete the questionnaires were given. HR recording was started (= 0 min), and the predetermined schedule began with the baseline rating (*t*_0_/$t_{0-VAS}$: VAS-A). Then (*t*_1_), the participants received instructions for the task (see Supporting information for the detailed instructions).

Right after, participants were requested to complete the questionnaires ($t_{2}∕t_{1-VAS}$: VAS-A). Then, the virtual session (*t*_3_) started. Participants entered the VE and had the chance to explore it briefly, for 90 seconds. This was the first time the participants learned about the topic of the speech, by reading the key points on the note on top of the lectern.

One and a half minutes after the immersion, a tone signalized the participants to begin their speech (*t*_4_) and after five minutes, a second tone chimed, signaling the end of the task. Participants removed the headset and immediately after this, the next rating was completed (*t*_5_/$t_{2-VAS}$: VAS-B). During a recovery period, further questionnaires were provided (*t*_6_: IPQ, cybersickness, VR experience, demographics). Last, the stress rating was filled out once again ($t_{7}∕t_{3-VAS}$: VAS-B). Afterwards, the HR band was removed. In a debriefing, the participants were informed that the objective of the procedure was to elicit a stress response. The chronological procedure and HR intervals are shown in Fig 4.

#### Participants.

Thirty-four participants were recruited from the University of Konstanz and the Konstanz University of Applied Sciences through the participant management platform SONA Systems® Uni Konstanz. The recruitment took place in February 2020. Each participant was randomly assigned to an experimental condition. There were no significant differences between the conditions for age and previous experience with VR.

Four of the participants were excluded post-hoc due to technical issues during their experimental session (*n* = 3) or disregard of the instructions (*n* = 1). This left a sample of 30 participants (21 females and 8 males), mean age 21.5 ± 2.4. All participants were informed about the nature of the experiment and were free to withdraw from it at any time. Participants were all German-speakers, had no neurological or psychiatric diagnosis, and wore no eyeglasses on the experimental session day (contact lenses were allowed).

### Results

#### Device comparison.

Presence, the experience of being actually in a Virtual Environment has been demonstrated to be key for behavioral realism [27]. Thus, a requisite for successfully replicating VR experiments designed for high-end devices in mobile VR is the comparability of experienced presence between devices. On the other hand, it is important to examine whether mobile VR experiments could elicit more cybersickness symptoms than high-end VR devices. This could be problematic in terms of participant dropout rates and behavioral outcomes. Therefore, we evaluated whether there was a difference in sense of presence or cybersickness from the public speaking experience depending on the device.

*Presence*. The sense of presence, measured using the IPQ, was analyzed by evaluating each of the IPQ subscales (realism, spatial presence, involvement) independently. Considering that previous VR experience could have an effect on the sense of presence, two-tailed Spearman correlations between each IPQ subscale and VR experience (“never", “1 to 5 times", “6 to 10 times", “11 to 15 times", “more than 15 times.") were carried out. There was a significant correlation detected for experienced realism, *r*(29) = −0.41,*p* = .028, but not for the other subscales (Spatial presence: *r*(29) = −0.11, *p* = .588; Involvement: *r*(29) = −0.17, *p* = .377) nor general presence, *r*(29) = −0.20, *p* = .289. Taking this correlation into account, we then conducted a ONEWAY ANOVA each with the presence subscales and, because of the detected correlation, experienced realism as dependent variables and the device as factor. No significant difference was detected between devices (general presence: F(1, 27) = 1.19, *p* = .286; spatial presence: F(1, 27) = 0.95, *p* = .340; involvement: F(1, 27) = 3.62, *p* = .068; experienced realism: F(1, 17.55) = 0.67, *p* = .423). Mean presence values can be seen in Table 2.

**Table 2:** Means and standard deviations of the presence subscales per device.

Device	IPQ subscale - M (SD)			
	General	Spatial presence	Involvement	Experienced realism
HTC Vive	3.6 (1.3)	2.6 (0.7)	3.9 (1.0)	3.0 (0.6)
Mobile VR	3.0 (1.8)	2.2 (1.2)	3.2 (1.0)	2.8 (1.1)

*Cybersickness*. Approximately 17% of all the participants experienced cybersickness symptoms due to VR. To examine device differences in cybersickness, a Fisher’s exact test was calculated for a 2 (yes/no occurrence of symptoms) x 2 (device) crosstab. No significant difference for the devices were found, *χ*^2^(1, N = 30) = 2.68, *p* = .157.

#### Stress response.

**Fig 5:**
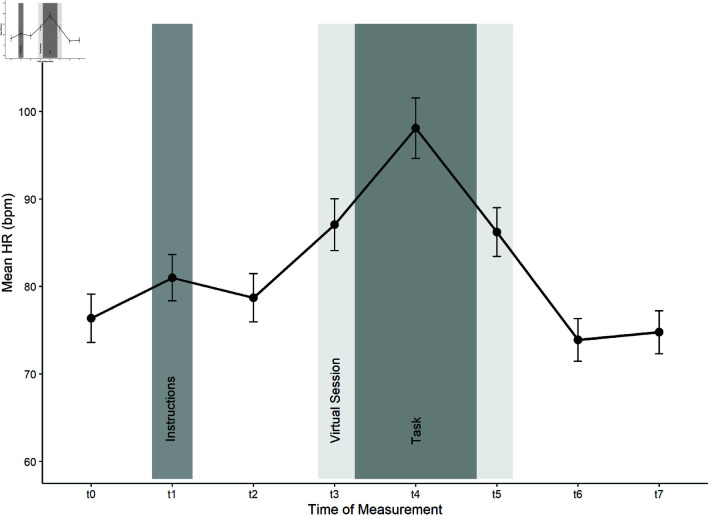
Course of the Heart Rate (HR). Course of the mean HR in beats per minute (bpm) per experimental interval ($T_{0}-T_{7}$).

**Fig 6:**
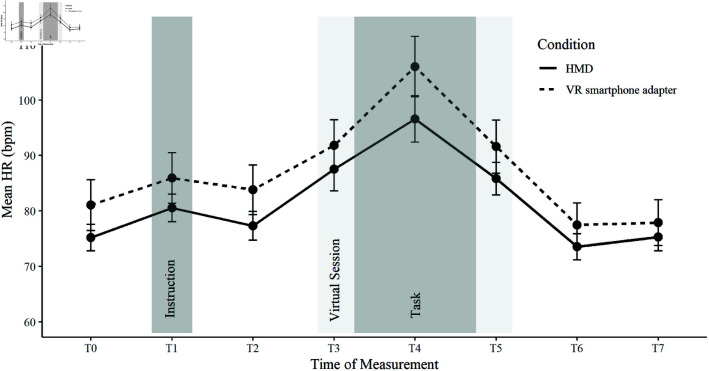
Course of the Heart Rate (HR) for factor device. Course of the mean HR in beats per minute (bpm) per experimental interval ($T_{0}-T_{7}$) and factor device.

To evaluate the physiological stress response from participants’ HR, an 8 (time: eight repeated baseline adjusted HR measures) x 2 (device) x 3 (audience) mixed factorial ANOVA was conducted to compare HR levels. The course of the mean HR can be observed in Fig 5 and separate for factor device in Fig 6. There was a significant main effect of time, *F*(2.17, 43.33) = 89.29, *p* < .001, $\eta_{part}^{2}$ = .817, *f* = 2.11. Bonferroni-adjusted posthoc analysis showed a significant difference (*p*<0.001) of *t*4to*t*0 (mean difference = 22.1, *p*<.001) indicating the HR response was due to the task. The HR score in *t*_4_ (*M* = 98.1 bpm, *SD* = 17.6) was the highest HR score, significantly different from the other time bins. There was no significant difference between t7 and t0 (mean difference = −1.94, *p* = .222) indicating a recovery of the HR back to baseline values. No statistical main effect was found for device, *F*(1, 20) = 0.05, *p* = .831 nor for audience, *F*(2, 20) = 0.14, *p* = .873. No significant interactions were detected (Time*device: F(2.17, 43.33) = 1.76, p = .182, Time*audience: F(4.33, 43.33) = 0.56, p = .706, Time*device*audience: F(4.33, 43.33) = 1.05, p = .395).

We evaluated the subjective post-task VAS rating regarding whether participants found the public speaking task stressful. A one-tailed one-sample t-test, with a reference value of 50 (0 = *do not agree*, 100 = *agree*) was conducted. The mean rating (*M* = 58.8, *SD* = 26.3) was significantly higher than the reference value, *t*(29) = 1.83, *p* = .039, *r* = .15.

### Discussion

The results show that in both the high-end VR device and the low-end mobile VR participants experienced physiological and subjective stress. Given that there was no significant difference detected regarding the type of audience in the virtual environment, following studies will be done utilizing the ‘attentive’ audience, where follows the participant with their gaze. Under the current conditions, no differences in presence or cybersickness were observed between devices. These findings provide preliminary evidence supporting the potential feasibility of using mobile VR for psychological experiments focused on specific tasks, such as stress induction and relaxation.

The next step investigating the use of mobile VR for field studies is to study the effect using mobile VR in different settings: in the laboratory, as it was done in Experiment 1, or in the field.

## Experiment 2

In the second experiment, we set out to investigate the effect of *setting* (laboratory vs. field) on the VR experiments. Further, we tested two different tasks, including the VR public speaking task used in Experiment 1. To select a second VE that was meaningfully different from the public speaking VE, we focused on three dimensions: *interaction level* between the environment and the participant, the *expected effect* in the participant, and the *medium* of the VE (i.e. whether it is computer generated or a video). Because the public speaking task is interactive, stress inductive and computer generated (CG), we decided to select a VE that is passive, inductive of mood improvement and is a 360 degrees video instead of CG. Given that previous studies have demonstrated that nature in VR is conductive of relaxation and mood improvement, in a similar manner than nature in real life [40], we opted for a mood improving nature environment as a second task.

### Method

#### Materials.

**Fig 7:**
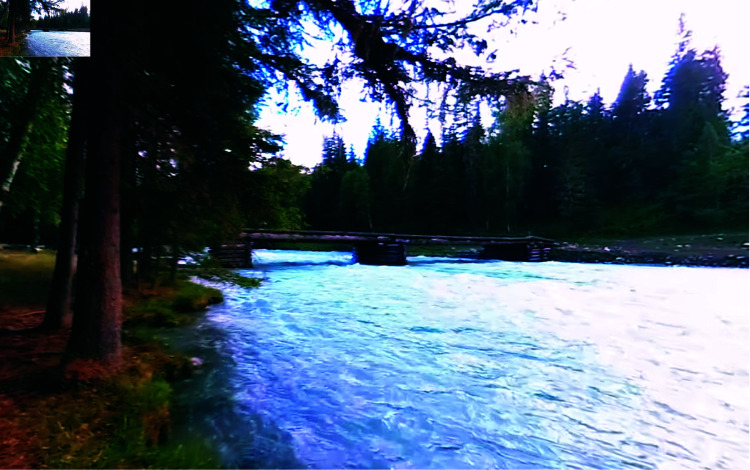
Still shot of the nature 360° video selected for Experiment 2 as second VE.

*Virtual environments*. We utilized the public speaking task described in Experiment 1 (using only the ‘attentive’ audience). As a second virtual environment for comparison, we utilized a publicly available 360° video of a natural setting. A still shot of the video can be seen in Fig 7.

According to Wang et al. [41], different types of video forest environments can have different effects on physiological stress and subjective mood states. After evaluating a set of 360° videos in a preliminary study (details in Supporting information), the video of a forest with a stream was selected (Meditation & Relaxation – Music Channel, 2019). The video consists of a forest with conifers, a flowing stream and includes some evidence of human presence in form of a bridge. The scene is accompanied by sounds of birds and water.

*Apparatus*. As a high-end commercial VR device, we utilized the HTC Vive Pro, as specified in Experiment 1. As low-end commercial VR, we utilized a previously manufactured Google Cardboard with a 90° FOV (Fig 1). Participants used their own compatible smartphone (limited in this case to Android OS) in both the laboratory and field conditions. Sound was provided by the participant’s own headphones. Each Google Cardboard was assembled by the participant. After the experiment, participants were able to keep it or dispose of it at their convenience.

#### Design.

Experiment 2 was conducted in a 3 (between: device setting) x 2 (within: task) mixed design. The device setting comprised the levels HTC Vive Pro in the laboratory (vive-lab), Google Cardboard in the laboratory (cardboard-lab), and Google Cardboard in the field (cardboard-field). Each participant completed the public speaking and nature tasks in a randomized order. The study was conducted in German. Participants received all the information, instructions, and questionnaires on a computer through WEXTOR® (https://wextor.eu [13,42]). Contact between the participants and the experimenter during the experimental phase was limited to interactions via the online video platform Zoom, regardless of the condition. In the laboratory conditions, the experimenter was in a separate room from the participant.

#### Tasks.

*Public speaking Task*. The public speaking VE from Experiment 1 was utilized. Some minor adjustments in the procedure were made: Participants were informed about the speech topic (‘Evolution’) together with the task instructions before entering the virtual environment, and were given 2 min time to prepare before their speech. In the cardboard conditions, the environment was run from the VR-mobile app that participants downloaded on their smartphones. In the HTC Vive Pro condition, the experience ran from the PC.

*Nature Task*. Participants were instructed to sit in a comfortable position and were informed that they would experience a nature environment. They were immersed in the VR, looked at the 360° nature video for 5 min, after which an alarm signalized the end. The video was played from YouTube in either the *Virtual Desktop app* for the HTC Vive Pro or the YouTube player in the participant’s smartphone.

#### Measures.

*Visual Analogue Scale (VAS)*. The VAS was used to repeatedly evaluate stress perception in the public speaking task. Participants were asked to rate the statement “I feel stressed.” or “During the task I felt stressed.” (VAS-B), on a line between “not at all” and “very much” (0-100, numbers not visible to the participant). The rating occurred at three different times during the experiment: at the beginning (*t*_0_, baseline), immediately after the task (*t*_1_, post-task, VAS-B), and after a recovery period (*t*_2_, recovery). Furthermore, for both, the public speaking and the nature task, VAS statements were used as control questions after the tasks (*t*_1_, VAS-control). For the public speaking task, the participant rated their satisfaction with their performance during in the speech they gave. For the nature task, the participant rated how much they liked the environment.

*Positive Affect Negative Affect Schedule (PANAS)*. The mood state before and after the nature task was measured using the Positive Affect Negative Affect Schedule (PANAS) originally developed by Watson et al. [43] . It consists of 20 adjectives, ten per primary mood dimension (positive or negative). Participants are asked to rate these items referring to their mood at the present moment. The intensity of feeling is graded between 1 (= very slightly or not at all) and 5 (= extremely) for each of the adjectives. The German version of the PANAS has demonstrated good internal consistency, with Cronbach’s *α* = .85 for Positive Affect (PA) and *α* = .86 for Negative Affect (NA) [44]. PANAS is a well-validated measure, frequently used in experimental contexts, and its application here ensures reliable assessment of mood changes induced by VR tasks.

*System Usability Scale (SUS)*. The System Usability Scale (SUS) by Brooke [45] was used to get an impression of the subjectively perceived handling of the devices and experiences. The questionnaire consists of 10 statements about, for example, the complexity of the system (“I found the system unnecessarily complex") and the requirement of prior knowledge for successful handling (“I needed to learn a lot of things before I could get going with this system"). The items are rated on a 5-point Likert scale with values between 0 (= strongly disagree) and 4 (= strongly agree). Previous research by Bangor et al. [46] demonstrated good internal consistency for the SUS, with Cronbach’s *α* = .91. Furthermore, the SUS has been shown to correlate highly (r = 0.91) with the Usability Metric for User Experience (UMUX) [47], which itself closely aligns with ISO 9241-11 usability standards, showing high sensitivity for slightly differences between systems. Given its robust psychometric properties and applicability to technology evaluations, the SUS was deemed appropriate for assessing the usability of both high-end and low-cost VR setups in this study.

*iGroup Presence Questionnaire (IPQ)* and *Cybersickness* were utilized as in Experiment 1.

#### Procedure.

As in Experiment 1, Experiment 2 was conducted in accordance with the ethical standards of the University of Konstanz and with the Helsinki Declaration. Informed consent was obtained from all patients who took part in the study. Participants were rewarded for their participation and they were explicitly made aware that they were allowed to withdraw from the study at any point in time, without losing their reward nor affecting the study.

For simplicity purposes, from here on we will refer to the public speaking task and its ratings as *Session A* and to the nature task and its ratings as *Session B*. As mentioned before, the order of the sessions was randomized per participant. The experiment started for both lab conditions with a quick personal welcome (ca. 2 min) to show the computer where all information would be presented. Participants first received the study information, gave their consent to take part in the experiment, and filled out demographic questions. After that, they were informed about the device they were going to use. Participants from both cardboard conditions were instructed to (a) download the VE using a link provided, and (b) build their cardboard from a package they received (which was shipped to participants from the cardboard-field condition in advance). A self-made video tutorial explaining how to assemble the cardboard was provided. Every participant received a guide with pictures on how to use and get immersed with their respective device.

Session A began with the baseline VAS rating on the subjectively perceived stress ($t_{0-A}$). Afterwards, the participants were instructed on the public speaking task. If there were no questions, after the 2-min preparation time, they started the experience and gave their improvised 5-min speech. They would then remove their headset and return to the computer for further instructions. Participants then rated one more time their subjective stress perception as well as their perceived task performance ($t_{1-A}$), and filled out questionnaires with regard to their experience with VR (QA: IPQ, sickness questions, SUS). To provide a similar recovery phase for every participant, after filling out the questionnaires, participants were asked to write a story about a topic of their own choice, for example the weather, an experience, or an invented storyline; the story should be exclusively for their own pleasure. They had 5 min to write this story. Lastly, participants completed the stress rating one last time ($t_{2-A}$) and were informed that the experimental session had finished.

Session B began with a baseline rating of the PANAS ($t_{0-B}$). Participants were instructed to sit down and simply experience the virtual environment. They started the experience and watched the nature video for 5 min. A tone signalized the end; participants took off their headset and returned to the computer for the second rating of the PANAS ($t_{1-B}$). They also rated on a VAS (VAS-control) to what extent they liked the virtual environment. As in Session A, participants completed questionnaires (*Q_B_*: IPQ, sickness questions, SUS), followed by writing a story for 5 min and finished the session with the last rating ($t_{2-B}$).

After these two sessions, participants filled out a question about their previous experience with VR. They received a debriefing as well as their allowance. For an overview about the procedure see S1 Fig.

#### Participants.

Sixty participants (39 females and 21 males, mean age 22.7 ± 2.7) were recruited from the University of Konstanz through the participant management platform SONA Systems® Uni Konstanz and the German National Research and Education Network (DFN) scheduler. The recruitment period took place between January and April 2021. Each participant was randomly assigned to an experimental condition. The nature task happened first for 31 participants. Due to technical problems one participant could not complete the public speaking task, thus their data was excluded from the analysis of this task (*N* = 59). There were no significant differences between the conditions for age, sex, previous VR experience, and the control ratings. Participants were all German-speakers, reported no neurological or psychiatric diagnosis, and wore no eyeglasses on the experimental session day (contact lenses were allowed).

### Results

Mixed analyses of variance (ANOVA) for main effects over time and planned comparisons for the interaction analyses of specific interest were conducted. From here on, the terms t0 to t2 are used to refer to VAS or PANAS ratings, for the public speaking task and the nature task respectively. To examine the time x condition interaction, the difference scores between the times were calculated ($t_{1}\;-\;t0$ = $\Delta t_{1}$, $t_{2}\;-\;t1$ = $\Delta t_{2}$). The baseline-corrected t1 (t1 - t0) and Δt1 (t1 - t0) are identical.

#### Presence.

Sense of presence was tested separately for both tasks. One-way ANOVAs were calculated with the presence subscales as dependent variables and the condition as group factor for both nature and speech tasks to examine the planned comparisons. However, it was first examined whether there is a relation between the previous VR experience and presence. Thus, a two-tailed Spearman correlation with previous experience was conducted for each IPQ subscale per task.

#### Public speaking task.

**Fig 8:**
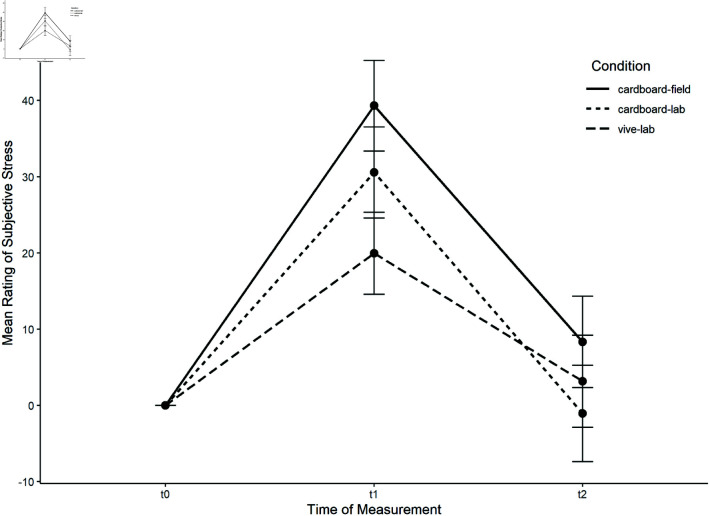
Course of the perceived stress per condition for the public speaking task.

A mixed ANOVA was calculated with the VAS stress rating as the repeatedly measured dependent variable, time ($t_{0}\;-\;t_{2}$) as within-subjects, and condition as between-subjects, factor. The course of perceived stress per condition can be observed in Fig 8. There was a significant main effect of time, *F*(2, 112) = 49.54, *p* < .001, $\eta_{part}^{2}$ = .469. Pairwise comparisons with a Bonferroni correction showed a significant difference between $t_{1}\text{and}t_{0}$ (*p* < .001) and $t_{1}\text{and}t_{2}$ (*p* < .001). No significant interaction between time and condition was detected, *F*(4, 112) = 1.91, *p* = .114.

Planned comparisons of the Δ-scores were then analyzed for stepwise interactions. First, a comparison between both lab conditions was conducted. For $\Delta t_{1}$ (change post-task in comparison with baseline) no significant difference was detected, *t*(56) = −1.31, *p* = .195. However, there was a difference for $\delta t_{2}$ (recovery), *t*(56) = 2.05, *p* = .045. In the following step, both cardboard conditions (lab and field) were compared. No significant difference was detected for $\Delta t_{1}$, *t*(56) = −1.07, *p* = .289 nor for $\Delta t_{2}$, *t*(56) = −0.08, *p* = .935. The results provide evidence that mobile VR in the field is conducive of stress induction, perhaps even more than in the lab conditions.

**Fig 9:**
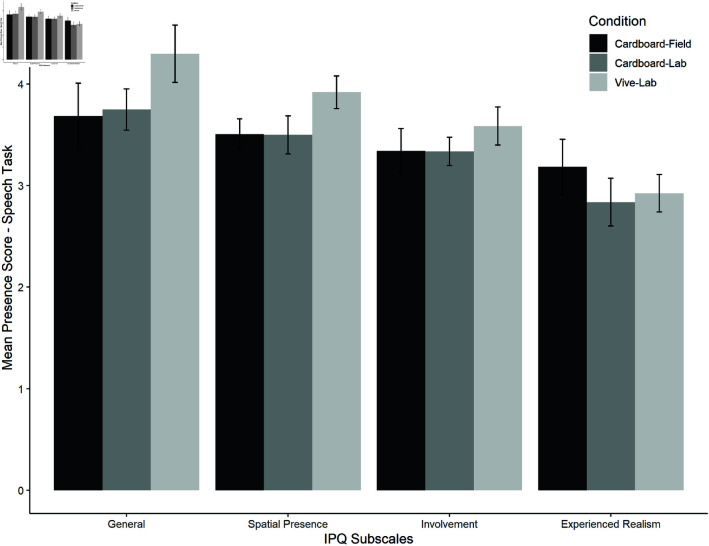
Mean presence ratings per subscale and condition for the public speaking task.

*Presence*. In the public speaking task, the Spearman correlations showed no significance for the presence and VR experience in this task (general presence: *r*(59) = 0.141, *p* = 0.144; involvement: *r*(59) = 0.089, *p* = 0.250; experienced realism: *r*(59) = −0.029, *p* = 0.412; spatial presence: *r*(59) = 0.076, *p* = 0.285). The planned comparison between both lab conditions and then between cardboard-lab and cardboard-field showed no significant differences between the presence scores (see Table 3 for details). Mean presence ratings per subscale are shown in Fig 9.

**Table 3:** Planned comparisons to examine differences between the conditions and the presence subscales (per task).

IPQ Scale	Hypothesis			
	H1		H2	
	t	p	t	p
Speech task				
General Presence	1.44	.156	0.17	.866
Involvement	0.97	.337	-0.02	.986
Experienced Realism	0.27	.789	-1.05	.297
Spatial Presence	1.78	.080	-0.02	.983
Nature task				
General Presence	0.00	>.999	-0.11	.915
Involvement	0.05	.960	-0.31	.761
Experienced Realism*^a^*	-1.16	.255	0.60	.553
Spatial Presence	0.78	.440	0.46	.649

H1 = comparison between vive-lab and cardboard-lab. H2 = comparison between cardboard-lab and cardboard-field.

*^a^* The Welch test was interpreted.

#### Nature task.

**Fig 10:**
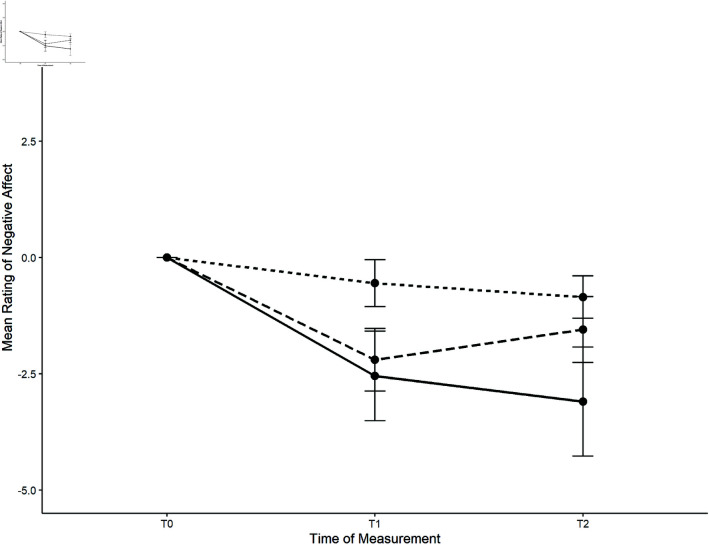
Course of perceived negative affect per condition for the nature task.

*Negative affect*. There was a significant main effect of time, *F*(1.44, 82.36) = 13.58, *p* < .001, $\eta_{part}^{2}$ = .192. Bonferroni-adjusted pairwise comparisons revealed significant differences between $t_{0}\text{and}t_{1}$ (*p* < .001) and $t_{0}\text{and}t_{2}$ (*p* = .001). The negative affect decreased by 2.2 (*SD* = 3.0) from the baseline to *t*_1_ in the vive-lab condition, by 0.6 (*SD* = 2.3) in the cardboard-lab, and by 2.6 (*SD* = 4.3) in the cardboard-field condition. No overall significant interaction between time and condition was detected, *F*(2.89, 82.36) = 2.00, *p* = .123. The course of perceived negative affect per condition is shown in Fig 10.

The planned comparison between the lab conditions showed no significant differences for $\Delta t_{1}$, *t*(57) = −1.58, *p* = .119, nor for $\Delta t_{2}$, *t*(57) = 1.52, *p* = .133. This was also the case for the comparisons between the cardboard settings for both Δ*t*_1_, *t*(57) = 1.92, *p* = .060, and Δ*t*_2_, *t*(57) = 0.40, *p* = .690.

**Fig 11:**
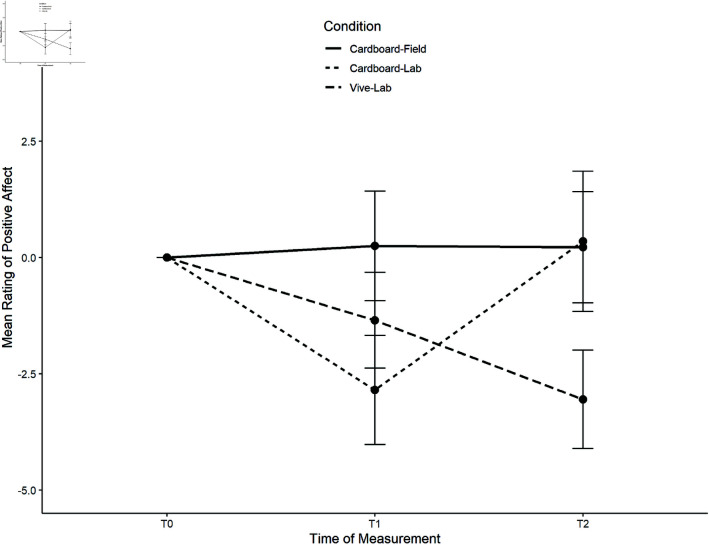
Course of perceived positive affect per condition for the nature task.

*Positive affect*. No significant main effect of time was detected, *F*(2, 114) = 1.95, *p* = .148. On average, the positive affect decreased after the task, there was, however, no significant differences between t1 and t0 (*p* = .144), t1 and t2 (*p* > .999), or t2 and t0 (*p* = .792). There was a significant time x condition interaction (*F*(4, 114) = 3.01, *p* = .021, $\eta_{part}^{2}$ = .095). The course of perceived positive affect per condition is shown in Fig 11.

Planned comparisons did not reveal any significant differences for Δ*t*_1_ between lab conditions, *t*(57) = 0.94, *p* = .351 or cardboard-lab and cardboard-field conditions, *t*(57) = −1.94, *p* = .057. There were, however, differences for Δ*t*_2_ between lab conditions, *t*(57) = −3.14, *p* = .003 and cardboard conditions, *t*(57) 2.07, *p* = .043.

**Fig 12:**
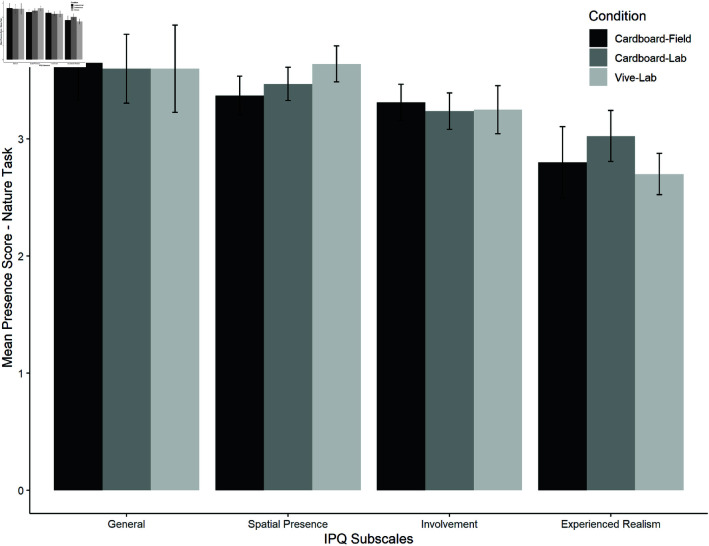
Mean presence ratings per subscale and condition for the nature task.

*Presence*. In the nature task, the Spearman correlations showed no significant relations for the presence and VR experience in this task as well (general presence: *r*(60) = −0.080, *p* = 0.272; involvement: *r*(60) = −0.215, *p* = 0.250; experienced realism: *r*(60) = −0.124, *p* = 0.173; spatial presence: *r*(60) = 0.003, *p* = 0.491). The planned comparison between both lab conditions and then between cardboard-lab and cardboard-field showed no significant differences between the presence scores (see Table 3 for details). Mean presence ratings per subscale are shown in Fig 12.

#### Cybersickness.

From the 59 participants, the cybersickness questions for *both* tasks (public speaking and nature) were answered completely by 53 participants. Thirteen participants out of these 53 overall valid cases reported sickness symptoms (six of them in both tasks). In the public speaking task, 11 participants reported an average of 1.6 (*SD* = 0.8) sickness symptoms; most of them experienced vertigo (*n* = 8). A Fisher’s exact test for the 2 x 3 crosstab containing the dichotomic variable “cybersickness occurred” (“yes" or “no"), and the different conditions, showed no significant difference of sickness occurring between the device settings *χ*^2^(2, *n* = 55) = 1.45, *p* = .584. In the nature task, an average of 1.8 (*SD* = 0.6) symptoms was reported by 11 participants. Most of them experienced vertigo (*n* = 8) and/or fatigue (*n* = 6). The Fisher’s exact test revealed no significant difference between the devices, *χ*^2^(2, *n* = 57) = 0.36, *p* = .917. The results suggest the comparable experience of cybersickness throughout the different device settings.

#### Usability.

**Fig 13:**
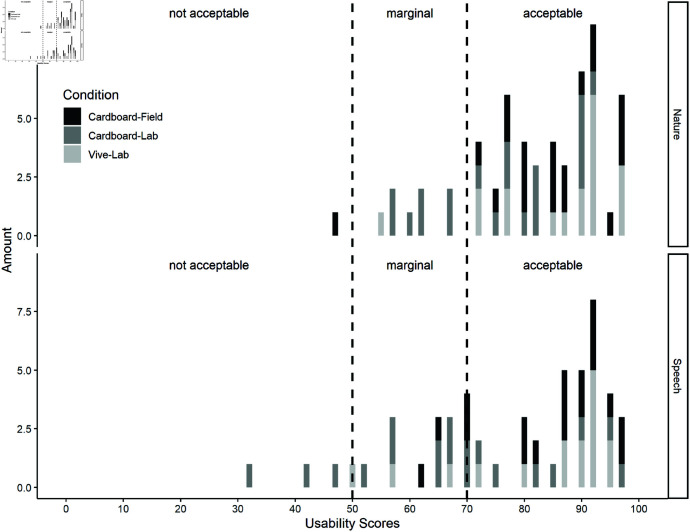
Individual SUS ratings per participant per task and condition.

The SUS results were examined descriptively. Mean scores for the usability were in general 81.8% (*SD* = 13.2), 82.6% (*SD* = 12.5) for the nature task and 80.5% (*SD* = 16.7) for the speech task (detailed mean scores and errors are shown in Table 4). The cardboard-lab condition received the lowest usability scores for both tasks. One out of 60 participants in the nature task (cardboard-field condition) and three out of 59 participants in the speech task (cardboard-lab condition) reported usability values below 50%, which is the threshold for non-acceptable usability scores. Participants’ individual SUS scores per task and condition can be seen in Fig 13.

## General discussion

Our findings indicate that VR experiments conducted in the field can elicit results comparable to those conducted in the lab under defined experimental conditions, supporting the feasibility of VR psychological field experiments. As expected, we found comparable results in both of our experimental tasks when carried out in the lab and in the field. Participants’ performance was overall comparable throughout the different conditions, as well as their subjective experience, as demonstrated by the usability results. In the particular case of stress induction, the results hint to a higher capacity of mobile VR in the field to induce stress in participants, however, further studies are necessary.

Unlike our original expectations, the laboratory condition did not have better *presence* outcomes than the field condition, regardless of potential distractors happening in the field condition and the difference in processing power and field of view between devices. One reason for this could be the emotional experience undergone by the participants. Previous research [22,48] has shown a relationship between emotion inductive VR experiences and enhanced presence. Thus, our results suggest that, in the context of psychological experiments, VR experience design and selection may be more relevant than experimental location and device selection.

Participants in the mobile VR conditions did not experience more cybersickness than with the HTC Vive Pro, as we had hypothesized. Overall, only 20% of the participants experienced any cybersickness symptoms. In comparison to previous studies [49], which report cybersickness in more than half of the participants, the occurrence in our study is rather low. This could be due to the lack of locomotion or navigation in our experimental tasks. Cybersickness often occurs when seen and experimented motions differ (sensory conflict) during the VR experience [50].

**Table 4:** Means and standard error for the SUS per task and condition.

	SUS - M (SE)		
Task	Vive-Lab	Cardboard-Lab	Cardboard-Field
Nature	87.75 (2.56)	75.88 (2.65)	84.13 (2.59)
Speech	86.75 (3.18)	69.63 (4.12)	85.26 (2.59)

The widespread availability and use of smartphones, combined with inexpensive cardboard VR devices, offers the potential to broaden access to psychological research by enabling remote participation and expanding the reach of studies beyond traditional laboratory settings. Taken together, our results provide preliminary evidence that VR can be a viable method for conducting psychological studies in field settings within specific experimental paradigms.

### General benefits of field VR studies

Beyond the capacity of using VR as a research tool outside of the laboratory, there are other benefits that come with VR field studies. First, mobile VR increases generalizability, given that each participant uses their own equipment, i.e. their own mobile phone. Avoiding limiting participants to a specific set of devices also decreases potential sampling effects [51]. In our study, we did not limit participation to a specific smartphone maker nor provided a specific Android mobile phone. Smartphones running the iOS can also utilize Google Cardboard.

Using mobile VR as the avenue for VR studies can potentially reduce self-selection effects. Unlike the alternative option of recruiting VR headset owners, cardboards can be shipped to a wider variety of participants, and these, at the same time, do not need to own a VR device or be knowledgeable about this technology.

Finally, the use of mobile VR can help increase the participant pool and reduce sample homogeneity, given that a cardboard can be shipped virtually anywhere, increasing the potential of recruiting diverse participant samples in different environments.

#### Low-cost mobile VR for laboratory studies.

Our results suggest that low-cost Cardboard devices can perform comparably to high-end VR systems on specific measures, such as subjective stress, demonstrating their potential as an accessible option for some VR-based psychological experiments. In our experiments, presence, a key element of VR experience, was experienced to a similar degree with the mobile VR. This finding is promising not just for VR field studies, but also for low-cost VR laboratory studies. Often, the high cost of a full VR set-up can make it difficult for laboratories to employ VR as a research tool. As seen in our results, it is even possible to obtain available materials online and utilize them for experimental purposes with the mobile VR, avoiding a second bottleneck for VR studies, which is the development of a 3D environment. Thus, it is possible to benefit from the special characteristics of VR without incurring in high costs.

### Considerations for running field VR studies

Based on our experience with VR experiments in the field, there are initial recommendations we can make for researchers who attempt to use mobile VR for field studies. It is useful to note that when combining field VR data with data from sensors on the smartphones used one has to consider the variations in these devices [52].

#### Availability through videoconference.

For comparability purposes between conditions, we carried all our experiments through videoconference, where we delivered the different electronic forms that contained instructions and questionnaires to the participants. During the experimental tasks, the participant was invited to turn off sound and camera for privacy purposes, increasing the chances of reducing demand effects. Even though the interaction with the participants was minimal, the experimenter was available for the participant throughout the experiment in case of questions or problems. This facilitated the flow of the experiments in the field and made it possible in our case to have a close-to-zero dropout rate throughout conditions (only one participant’s data had to be excluded due to technical issues). Indeed, the final goal of VR field experiments would be to have a fully unsupervised set-up, it is recommended to utilize the videoconference format for initial trials or in experiments where it is possible and meaningful to have this blended set-up.

#### Shipping the cardboards.

Unassembled cardboards were shipped to a postal address given by the participant at the time of registration for the experiment (which happened online). In a pilot study, pen and pencil questionnaires were also sent to participants together with a prepaid return envelope to ship the answers back to the experimenters. In cases in which it is not possible for the participants to access online forms, it is also possible to utilize this format. Given that a stable Internet connection and a computer were basic requirements for our experiment (because of the video conference), we made use of WEXTOR (https://wextor.eu [13]) for the questionnaires instead of paper questionnaires and avoided the need for participants to ship back their questionnaires, which we hypothesize may be another step likely to increase the dropout rate.

#### Assembling the cardboards.

Participants received a video with a visual explanation on how to assemble the cardboard. Despite this, some of the participants struggled with the assembly process and requested help by the experimenter via the video call. Lack of such support can potentially become another dropout cause in unsupervised field experiments. In future studies, it is necessary to experiment and investigate different solutions to improve the cardboard assembly task. It is important to remark that it is also possible to build a cardboard from scratch with a small set of easy-to-find materials. This alternative can also be explored in the future in cases where it may be meaningful.

### Limitations

#### Interactivity with mobile VR.

One pitfall of using Google Cardboard is the lack of basic interaction devices, such as controllers or extra trackers, which are included by default in more sophisticated VR equipment. A typical interaction method for Cardboard is the use of ‘gaze’ measured by head position (since there is in general no eye-tracker directly available in the mainstream smartphones). This method can, however, be straining for the user and disturb interactive tasks.

This lack of readily available interaction methods means that the variety of tasks that can currently be used with mobile VR are somewhat restricted. Several experimental interaction methods have been developed for cardboard, however, they are not readily available yet (e.g. [53]). Thus, in future studies, it will be necessary to explore and test different interaction possibilities and find the most versatile and effective for VR field studies.

#### Selected tasks.

Even though our study included tasks spanning three dimensions—interaction level, media, and participant effects—it is important to acknowledge that several experimental features remain untested. For example, tasks requiring navigation within the virtual environment or those involving participant embodiment in the VE were not included in our experiments. These untested features represent critical areas for future research, as task-specific requirements, such as locomotion, embodiment, or others, may significantly influence the feasibility and effectiveness of mobile VR in field settings. Consequently, our findings should be interpreted as initial evidence within a limited scope of experimental paradigms, underscoring the need to evaluate mobile VR across a broader range of tasks and dimensions in order to be able to determine its generalizability to various types of psychological experiments.

#### Population.

In our study, all our participants were young and technologically versed. It is yet to be investigated what are the possible problems and solutions of use of VR in the field for people of different age groups and technological literacy levels. Furthermore, a pre-requirement for mobile VR is for the participant to have access to a smartphone. Potentially, a cheap smartphone could be shipped to the participants but this would increase the complexity of the set-up for the user. Smartphone-based field studies are feasible and cross-checking of quality of data from such devices is possible (e.g. [54]).

## Conclusion

Virtual Reality is a promising tool for psychological research, though VR field experiments are still relatively uncommon. In this study, we have provided preliminary evidence supporting the feasibility of using VR as a medium for specific psychological field experiments, such as stress induction and relaxation tasks, and demonstrated that low-cost VR equipment can be effectively employed in both laboratory and field settings. We envision that our findings will help advance the use of VR for field-based assessments and applications, paving the way for broader adoption in psychological research.

## Supporting information

### Experiment 1

#### Speech instructions.

The following are the written instructions the participant received before the speech task in Experiment 1. Instructions were provided in German: *“Soon, you will go into virtual reality. You will be situated in a conference room. You can first have a look around, if you want to. On a lectern in front of you, there will be a note with three written speech key points on it. Your task is to hold a five-minute speech about the topic of these key points. It is up to your choice if you include all three, two, or just one key point in your speech. Try to complete the task to the best of your ability. Your speech is going to be recorded on audio. Please start your speech when you hear the audio signal (a ’beep’). You are going to hear a second signal when your time, the five minutes, is over. Please try to speak for the whole time. Try to the best of your ability and be willing to get involved in the virtual environment. Do you have any questions?"*

### Experiment 2

S1 Fig Procedure in Experiment 2. Ratings for the stress task: VAS, for the nature task: PANAS, b: VE = virtual environments, c: IPQ, sickness, SUS.
(TIF)

#### Preliminary study for the 360° video selection.

To choose an appropriate 360° nature video as second task for Experiment 2, we first pre-selected five publicly available videos that depict a forest including nature sound, varying in including water elements (a stream or waterfall) or signs of human presence (a bridge). The final collection was composed by these five nature videos and three videos with other content to mask the intention of the study (tornado, roller coaster, traffic jam). Still shots of this set of 360 videos can be seen in S2 Fig. A pilot study was conducted to select the 360° video with the highest valence and lowest arousal. Ten participants rated the seven 360° videos in the collection. To measure valence and arousal a Visual Analogue Scale reaching from 0-100 was used for each dimension per video. The video showing a forest and a stream (B, in S2 Fig) was rated to have the highest valence (*M* = 90.95, *Median* = 96.25, *SD* = 11.99) and lowest arousal (*M* = 14.05, *Median* = 2.00,* SD* = 27.38). Furthermore, it showed a lower variance for both valence and arousal in comparison to the second-best rated video, indicating consistency across the participants.

S2 Fig Still shots of the set of 360 videos used in the preliminary study. F-H are the videos used in the study. Links to the videos: A: https://www.youtube.com/watch?v= jYLArug2nsU (Forest with wind). B: https://www.youtube.com/watch?v=YFWCsiJjaq0 (At a sparkling stream). C: https://www.youtube.com/watch?v=0Ai-iKQEnQY (Waterfall). D: https://www.youtube.com/watch?v=VP9tyky7SBQ (Bridge with water). E: https://www.youtube.com/watch?v=8-WpK_Lyr_Q (Pine forest). F: https://www.youtube.com/ watch?v=TAZDxjPMxLc (Traffic jam). G: https://www.youtube.com/watch?v=V5rJVPSSoFs (Rollercoaster Canada without screaming). H: https://www.youtube.com/watch?v=b07EL3Mf7eg (Tornado).
(TIF)
