# Supplementary figures and images for: Virtual Reality experiments in the field
Source: PLoS One. 2025 Apr 8;20(4):e0318688. doi: 10.1371/journal.pone.0318688 (PMC11978061; doi:10.1371/journal.pone.0318688)

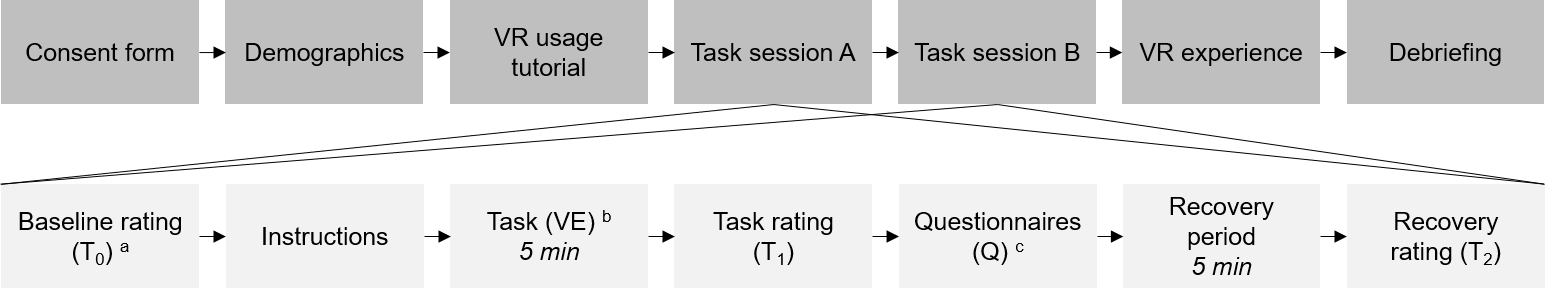

Supplement: S1 Fig — Ratings for the stress task: VAS, for the nature task: PANAS, b: VE = virtual environments, c: IPQ, sickness, SUS. (TIF) [file pone.0318688.s001.tif]

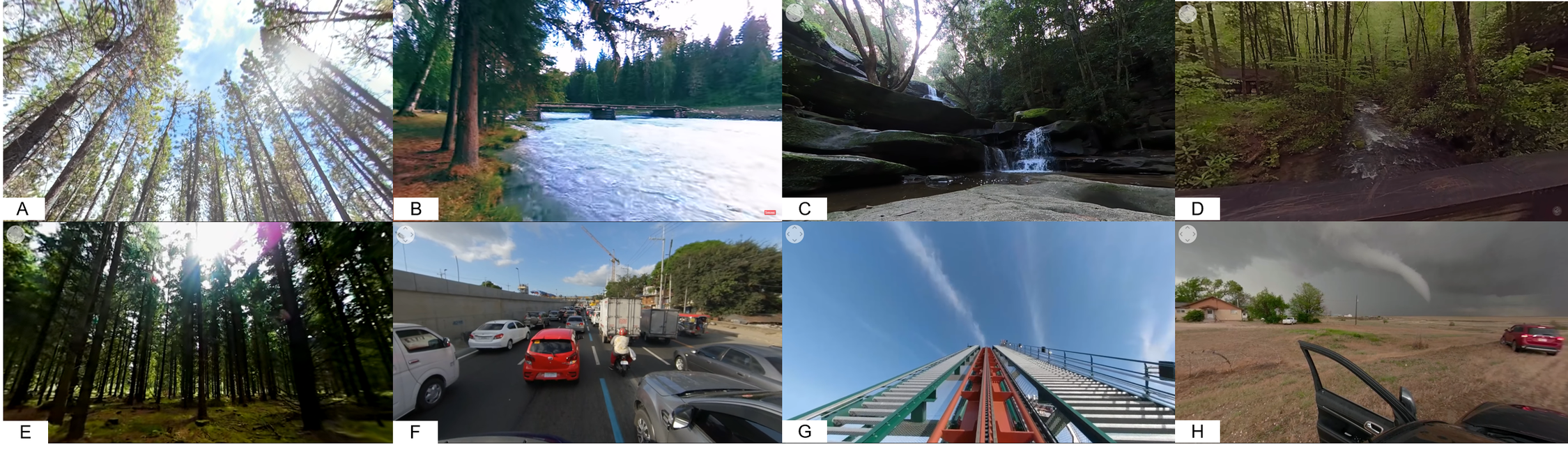

Supplement: S2 Fig — F-H are the videos used in the study. Links to the videos: A: https://www.youtube.com/watch?v= jYLArug2nsU (Forest with wind). B: https://www.youtube.com/watch?v=YFWCsiJjaq0 (At a sparkling stream). C: https://www.youtube.com/watch?v=0Ai-iKQEnQY (Waterfall). D: https://www.youtube.com/watch?v=VP9tyky7SBQ (Bridge with water). E: https://www.youtube.com/watch?v=8-WpK_Lyr_Q (Pine forest). F: https://www.youtube.com/ watch?v=TAZDxjPMxLc (Traffic jam). G: https://www.youtube.com/watch?v=V5rJVPSSoFs (Rollercoaster Canada without screaming). H: https://www.youtube.com/watch?v=b07EL3Mf7eg (Tornado). (TIF) [file pone.0318688.s002.tif]
